# Toward the use of CVD-grown MoS_2_ nanosheets as field-emission source

**DOI:** 10.3762/bjnano.9.160

**Published:** 2018-06-07

**Authors:** Geetanjali Deokar, Nitul S Rajput, Junjie Li, Francis Leonard Deepak, Wei Ou-Yang, Nicolas Reckinger, Carla Bittencourt, Jean-Francois Colomer, Mustapha Jouiad

**Affiliations:** 1 Department of Mechanical and Materials Engineering, Masdar Institute of Science and Technology, A part of Khalifa University of Science and Technology, 54224, Abu Dhabi, United Arab Emirates; 2 Research Group on Carbon Nanostructures (CARBONNAGe), University of Namur, 61 Rue de Bruxelles, 5000 Namur, Belgium; 3 Department of Advanced Electron Microscopy, Imaging and Spectroscopy, International Iberian Nanotechnology Laboratory (INL), Avenida Mestre Jose Veiga, Braga 4715-330, Portugal; 4 Engineering Research Center for Nanophotonics & Advanced Instrument, Ministry of Education, School of Physics and Materials Science, East China Normal University, 3663 North Zhongshan Road, Shanghai 200062, China; 5 Chimie des Interactions Plasma-Surface (ChIPS), CIRMAP, Research Institute for Materials Science and Engineering, University of Mons, Mons, Belgium

**Keywords:** chemical vapor deposition (CVD), field emission, molybdenum disulfide (MoS_2_), nanosheets, sulfurization, transmission electron microscopy (TEM)

## Abstract

Densely populated edge-terminated vertically aligned two-dimensional MoS_2_ nanosheets (NSs) with thicknesses ranging from 5 to 20 nm were directly synthesized on Mo films deposited on SiO_2_ by sulfurization. The quality of the obtained NSs was analyzed by scanning electron and transmission electron microscopy, and Raman and X-ray photoelectron spectroscopy. The as-grown NSs were then successfully transferred to the substrates using a wet chemical etching method. The transferred NSs sample showed excellent field-emission properties. A low turn-on field of 3.1 V/μm at a current density of 10 µA/cm^2^ was measured. The low turn-on field is attributed to the morphology of the NSs exhibiting vertically aligned sheets of MoS_2_ with sharp and exposed edges. Our findings show that the fabricated MoS_2_ NSs could have a great potential as robust high-performance electron-emitter material for various applications such as microelectronics and nanoelectronics, flat-panel displays and electron-microscopy emitter tips.

## Introduction

There is a great interest in the development of one- and two-dimensional (1D and 2D) materials for field-emission (FE) based cathodes using various nanostructured materials [1] for applications in displays, X-ray sources and cold-cathode electron sources [2]. 1D and 2D materials such as carbon nanotubes [3], ZnO nanorods [1], LaB_6_ nanowires [2], SnS_2_ nanosheets (NSs) [4], vertically aligned graphene [5], WS_2_ nanotubes [6], MoSe_2_ nanosheets [7], and MoS_2_ NSs [8–10] are potential field-emitter candidates. The FE properties depend on the microstructure of the materials, such as morphology, orientation, size and internal or intrinsic features [1]. Among the different morphologies of 1D and 2D materials, vertically aligned nanostructures are considered as good candidates for field emission. Due to their exposed sharp edges, un-stacked morphology and high aspect ratio they are less affected by Joule heating [11]. In the past few years, FE measurements on different MoS_2_ morphologies, such as horizontally arranged (with a few protruding portions) MoS_2_ [12], sparsely distributed vertically aligned MoS_2_ NSs [9], MoS_2_ nanoflowers [13] and MoS_2_ nano-heteroarchitectures [14] have been reported. The semiconducting MoS_2_ NSs with exposed edges could significantly enhance the FE properties [9]. It is well known that the electrical and optical properties of MoS_2_ are influenced by their size, shape [15–16] and the number of layers [16]. Various methods have been used to synthesize vertically aligned MoS_2_ NSs: liquid-phase exfoliation [17], hydrothermal synthesis [8] or chemical vapor deposition (CVD) [15,17–18]. CVD is regarded as the most promising method to synthesize high-quality MoS_2_ with good control over size, shape and morphology [17,19]. So far, relatively few FE measurements on vertically aligned MoS_2_ NSs [9] and nanoflowers [13] have been reported. Significant challenges still remain in the development of MoS_2_ nanostructures for large-scale FE devices using a simple, efficient and low-cost production technology with high quality and large quantities. An ideal FE material would have a low work function, aligned arrays of sharp tips, large aspect ratio, high stability and moderate current density, as well as the capability to be placed easily on a conductive substrate [1]. Herein, we report on the FE properties of densely packed and uniformly distributed vertically aligned 2D MoS_2_ NSs, well adhered to the substrate. These NSs were synthesized by double sulfurization of sputter-deposited Mo films on Si (300 nm SiO_2_/Si) substrates. The FE properties assessment is carried out on the NSs transferred onto a conducting fluorine-tin-oxide (FTO) substrate.

## Experimental

### Sample preparation

The NSs were grown on SiO_2_/Si substrates via double sulfurization of a sputter-deposited 50 nm Mo film using an ambient-pressure CVD technique. Flushing of the quartz tube using Ar gas stream, followed by continuous Ar flow for 1 h was performed. This reduces the oxygen content in the reactor prior to the sulfurization process. The growth conditions were taken and improved from our previously reported MoS_2_ NS synthesis method [18]. In a typical sulfurization process, 220 g sulfur powder was placed at the 40 °C temperature zone and the 850 °C temperature zone (total 440 g) along with the Mo film sample placed at the 850 °C temperature zone in the downstream of the Ar flow in the quartz tube reactor. In the first sulfurization step of 30 min the S powder was placed at the 800 °C zone. The optimized second sulfurization step was performed by inserting the quartz tube in the hot zone of the furnace, such that the S powder placed at 40 °C reaches the 400 °C temperature zone. The sample remained at 850 °C as the quartz reactor was moved over a few centimeters.

### Wet chemical transfer

A polymer-assisted wet-chemical method was employed to transfer the MoS_2_ layer on conducting substrates [20]. A layer of poly(methyl methacrylate) (PMMA), 200 nm thick, was coated onto the surface of the MoS_2_/SiO_2_/Si sample surface (PMMA/MoS_2_/SiO_2_/Si), then floated on buffered oxide etchant. After leaving it overnight, the silica layer was removed, freeing the PMMA/MoS_2_ film from the growth substrate (SiO_2_/Si). The sample was subsequently transferred to deionized water to rinse the chemical etchants. Then, the desired substrate (here, FTO) was used to lift the PMMA/MoS_2_ out of the water. The sample was then dried overnight to let the water trapped underneath the MoS_2_ NSs to be removed naturally. Next, the sample was baked at 110 °C for 10 min to improve the uniformity and the adhesion to the substrate. Finally, the PMMA was dissolved in acetone.

### Physical characterization methods

The morphology of sulfurized Mo films on SiO_2_/Si substrates was characterized using field-emission scanning electron microscopy (FE-SEM) combined with a Helios FEI^TM^ NanoLab 650 focused ion beam (FIB) system. Transmission electron microscopy (TEM) lamella were prepared using the standard FIB lift-out technique described in an earlier report [21]. To have a plane view of the deposited material, the sample was locally capped using FIB-assisted Pt deposition and the cut block was lifted out using an Omniprobe^TM^. The block was tilted at 90° relative to its original position and mounted onto a TEM grid. The TEM investigations were performed using an image-corrected Titan G2 FEI^TM^ system. For selected samples, cross-section TEM analyses were carried out using an aberration-corrected FEI^TM^ Titan ChemiSTEM system (equipped with a Cs probe corrector, a high-angle annular dark-field imaging (HAADF) detector and a four quadrant Super X energy-dispersive spectroscopy (EDS) detector) operating at 200 kV for imaging and elemental characterization. Roughness and topography of the as-grown MoS_2_ NSs (before transfer) were examined by atomic force microscope (AFM). The AFM scans were recorded in resonant mode (AppNano^TM^ made cantilever with tip radius below 10 nm) with a resonant frequency of 312 kHz. To confirm the layer number of the NSs, micro-Raman spectroscopy was performed using a 473 nm laser at room temperature. X-ray photoelectron spectroscopy (XPS) measurements were performed using a Thermo Fisher Scientific K-alpha spectrometer with a 250 μm diameter X-ray spot. The FE properties of the MoS_2_ NSs film transferred on the conductive FTO glass substrate (Figure 5) were measured using a custom-built conventional diode-type structure over a 1 cm^2^ area in a chamber under high vacuum (4.0 × 10^−6^ mbar). The thin film of MoS_2_ NSs deposited on the FTO glass substrate served as electron-emission cathode and another piece of conductive FTO glass was used as anode. The distance between the cathode and anode was fixed at 220 µm by using thin glass spacers. The FE current (*I*) versus the applied voltage (*V*) was measured using an electrometer (Tianjin Dongwen, China) and a high-voltage direct current power supply. The FE current stability was investigated using a computer-controlled data acquisition system with a certain sampling interval.

## Results and Discussion

### MoS_2_ NSs morphology and composition

**Figure 1:**
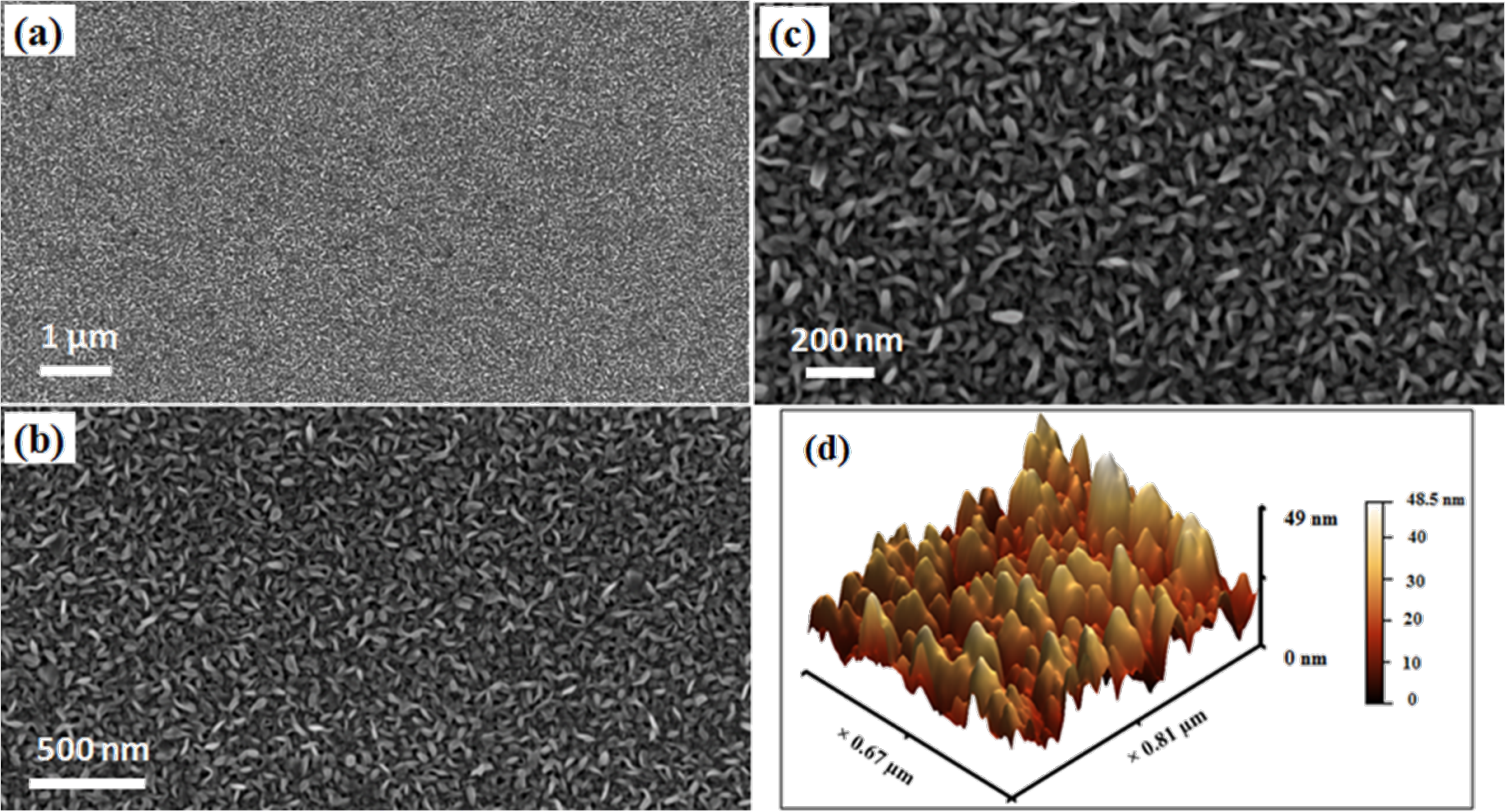
(a**–**c) Typical field-emission SEM images with different magnifications of MoS_2_ NSs grown by double sulfurization of a 50 nm Mo film at 850 °C on SiO_2_/Si substrates; (d) AFM image: 3D image of panel (b) in Figure S1.

The continuous and dense distribution of vertically aligned MoS_2_ NSs on SiO_2_/Si substrates with a thickness of 5–20 nm can be observed from the representative FE-SEM image shown in Figure 1. An optimized double sulfurization step gives a homogeneous distribution of MoS_2_ NSs over the entire sample surface without forming any aggregated crystals. Therefore, unlike in the previous case [20], the growth of micrometer-size crystals (of Mo or MoO_3_) on the sample surface was not observed. The AFM measurements confirm (Figure S1, Supporting Information File 1) a dense growth of MoS_2_ NSs. The observed root mean square roughness was 8 nm (measured over an area of 2.5 × 2.5 µm^2^). The 3D image (Figure 1d) confirms a tip-like morphology of the MoS_2_ NSs, which is believed to possess an important role in the measured FE current (discussed later). It can also be noted that the growth of MoS_2_ NSs reported here is of better quality in terms of homogeneous distribution and vertical alignment compared to the previously reported MoS_2_ nanostructures (on which FE studies were performed), which were sparsely and randomly distributed [9]. The NSs reported by Kashid et al. have few protruding MoS_2_ NSs and mostly planar surfaces [12].

**Figure 2:**
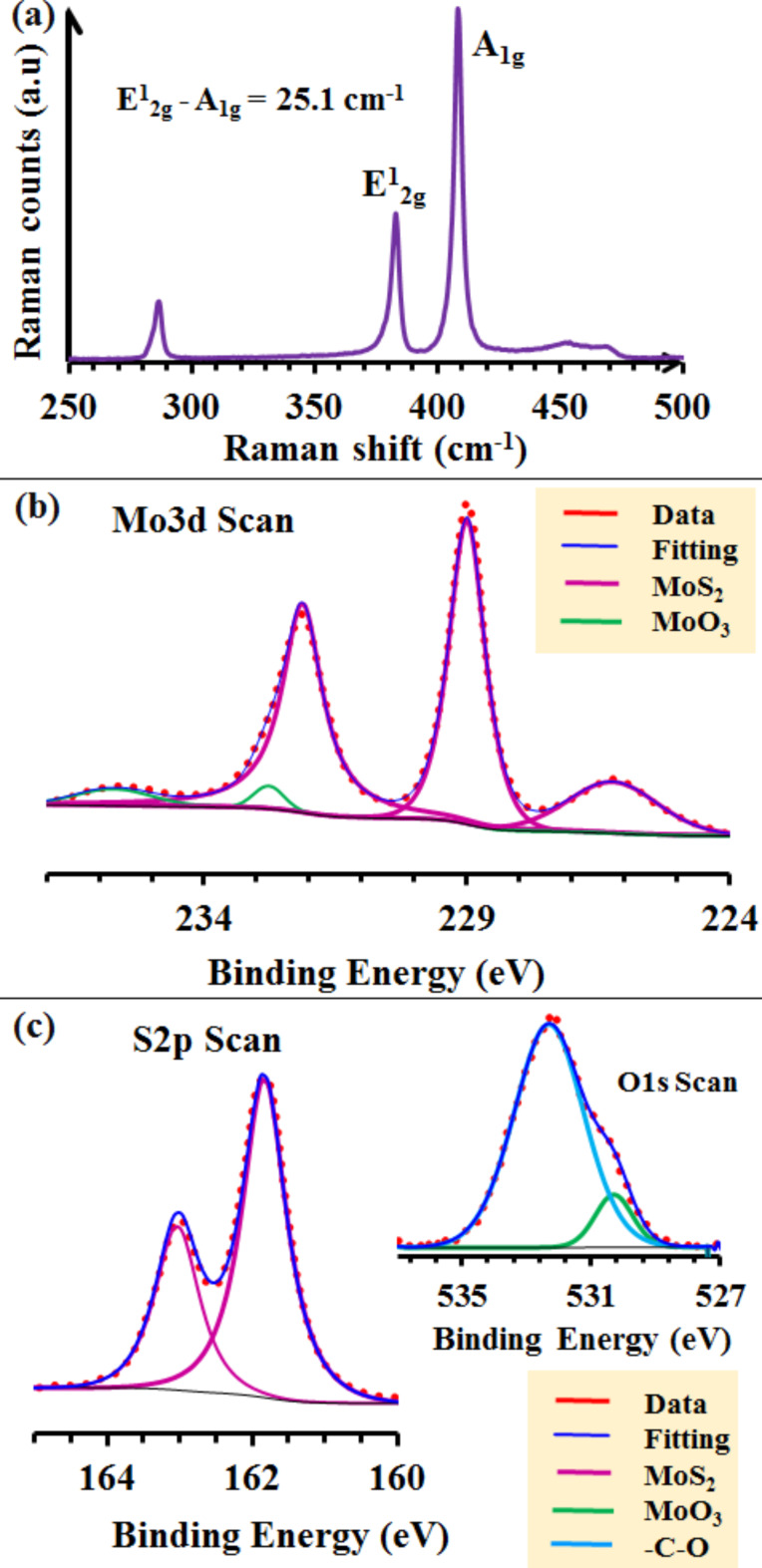
MoS_2_ NSs grown by double sulfurization of a 50 nm Mo film at 850 °C on SiO_2_/Si substrates: (a) typical micro-Raman spectrum; (b) Mo 3d core level and (c) S 2p core level with, in the inset, the O 1s core-level spectrum. The doublet peaks marked with identical color correspond to one phase. More detailed information is summarized in Table S1 (Supporting Information File 1).

A typical Raman spectrum of the as-synthesized MoS_2_ NSs, is shown in Figure 2a, indicating the characteristics of the 2H-MoS_2_ in-plane vibrational mode (E^1^_2g_) at 383.1 cm^−1^ and the out-of-plane vibrational mode (A_1g_) at 408.3 cm^−1^ [22]. The difference in frequency between the two vibration modes is 25.2 cm^−1^, which indicates the presence of more than three layers of MoS_2_ [23]. The chemical state of the as-grown samples was investigated by XPS. The Mo 3d, S 2p and O 1s high-resolution core-level spectrum fits are presented in Figure 2b,c. The corresponding data analysis results are given in Table S1 (Supporting Information File 1). In the high-resolution Mo 3d core-level spectrum fit, the doublet Mo 3d_5/2_ and Mo 3d_3/2_ peaks at 228.9 and 232 eV, respectively, are attributed to the formation of MoS_2_ (Figure 2b) [23–24]. This is further confirmed by the presence of a shoulder in the S 2s region at 226.2 eV [23]. A very small contribution corresponding to MoO_3_ (Mo^6+^ oxidation state) phases was also observed (Figure 2b) with Mo 3d_5/2_ and Mo 3d_3/2_ component peaks at 232.7 and 235.6 eV, respectively [24]. Additionally, the presence of the main MoS_2_ phase (corresponding to the observed major peaks) is confirmed by the S 2p core-level spectrum fit presented in Figure 2c, with the S 2p_3/2_ and S 2p_1/2_ component peaks appearing at 161.8 and 163 (MoS_3_) eV (Table S1, Supporting Information File 1), respectively, with a spin–orbit energy separation of 1.2 eV corresponding to MoS_2_ (S^2−^ oxidation state) [24]. In the O 1s core-level spectrum (inset of Figure 2c), a small peak corresponding to MoO_3_ in agreement with the Mo 3d spectra fitting with a binding energy of 530.2 eV was observed [24–25]. The other peak at 532.3 eV is assigned to O–C bonds [25]. This peak could be explained by the fact that the sample was stored in air for several days before performing the XPS measurements. Thus, the surface-sensitive characterization technique (XPS) shows the dominant presence of the MoS_2_ phase on the sample surface.

### Microstructural analysis of the MoS_2_ NSs

**Figure 3:**
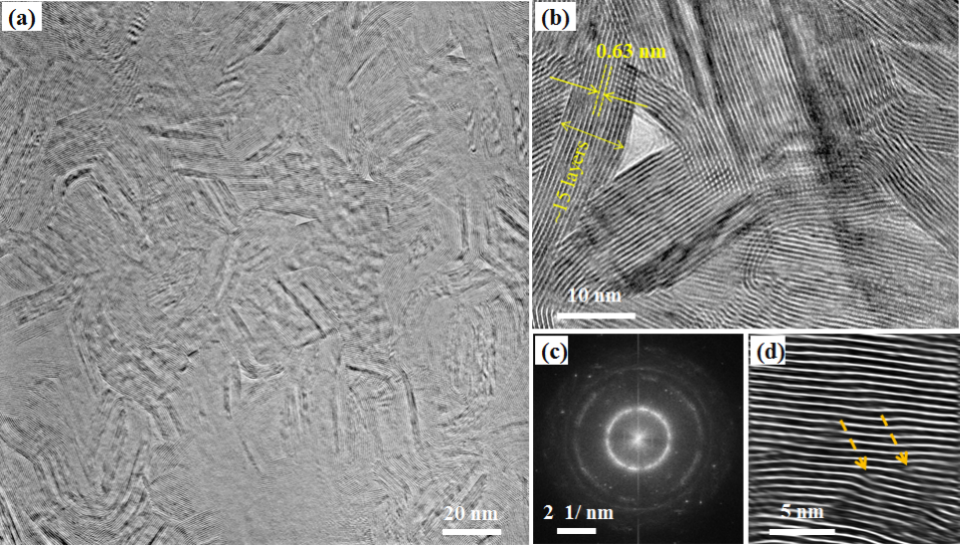
MoS_2_ sample grown by double sulfurization of a 50 nm Mo film at 850 °C on SiO_2_/Si substrates: (a) Plane-view HRTEM image; (b) high-magnification TEM image; (c) FFT pattern of panel (a); (d) filtered HRTEM image indicating the presence of sheet stacking defects (indicated by orange arrows).

In Figure 3, plane-view images of as-grown MoS_2_ NSs are presented. A bright -field TEM image (Figure 3a) indicates the presence of NSs over the entire area. The as-grown NSs, densely packed with very high crystalline quality, can be seen. The fast Fourier transform (FFT) image of Figure 3a is given in Figure 3c. The ring pattern indicates that the NSs are made of MoS_2_ polycrystals. A high-resolution (HR) TEM image is shown in Figure 3b. The stacking periodicity (the interlayer distance) is found to be around 0.63 nm. The number of layers in a NS is found in the range of 15–20. One should note that the NS thickness cannot be solely determined by plane-view TEM since some layers could be viewed via the bended NSs. Figure 3d is a filtered HRTEM image showing evidence of MoS_2_ NS stacking defects highlighted by the arrows. These defects are inherent to the fabrication process. This NSs stacking configuration could exhibit interesting properties in membrane technologies such as filtration membranes to remove fouling, heavy metals and chemicals from water by membrane separation as reported elsewhere for graphene nanosheets [26].

**Figure 4:**
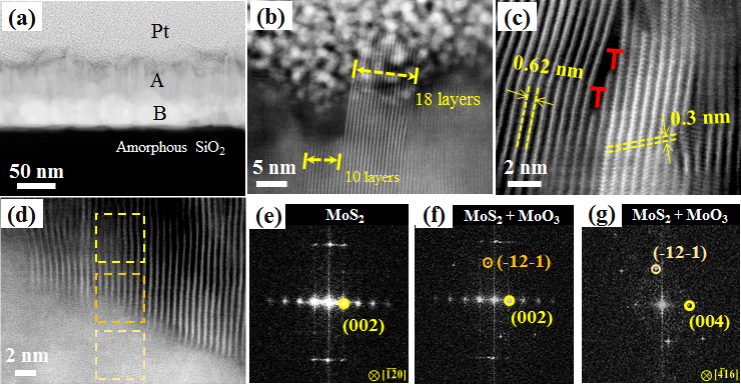
MoS_2_ sample grown by double sulfurization of a 50 nm Mo film at 850 °C on SiO_2_/Si substrates: (a) HAADF-STEM image at low magnification with different observed materials layers marked; (b) TEM image at the interface between the MoS_2_ NSs and the Pt layer; (c) higher magnification of layer A showing edge dislocation (marked with “T”) in the MoS_2_ layers; (d) high-magnification HAADF-STEM image at the interface between “A” and “B” from panel (a); (e–g) FFT analysis over the area marked by the dotted squares in panel (d), from top to bottom respectively.

To investigate further the NSs growth, cross-section TEM measurements were performed. The general morphologies of the vertically standing and densely packed MoS_2_ NSs grown on SiO_2_/Si substrates can be seen from the low-magnification scanning TEM (STEM) image (Figure 4a). The STEM image (Figure 4b) demonstrates that the MoS_2_ NSs growth occurred perpendicular to the substrate. The height of the NSs ranges from 50 to 70 nm. The corresponding layer is marked with “A” in Figure 4a. Each NS consists of 10 to 20 MoS_2_ layers. From Figure 4b, detailed structures at the tip of the MoS_2_ NSs can be observed. It reveals the presence of NSs with exposed edges, which may act as emission sites. The active sites of MoS_2_ NSs edges are catalytically active and are thus highly preferable as a catalyst surface over the relatively inert MoS_2_ basal plane [27]. Figure 4c shows the atomic structure of the MoS_2_ NSs with some edge dislocations (labelled as “T”) along the *c*-axis. Moreover, the interplanar distances are ca. 0.62 and ca. 0.30 nm, corresponding to the (002) and (004) planes of 2H-MoS_2_ [18]. However, a slightly higher interplanar distance of 0.63 nm near the edges (in agreement with plane-view TEM) was also observed. It indicates that the NSs possess a slightly different lattice parameter due to the crystal confinement at the top end. Additionally, the FFT pattern as shown in Figure 4e for the yellow squared area in Figure 4d, confirms well-crystallized MoS_2_ NSs with the *c*-axis being normal to the NSs. Between, the vertically aligned MoS_2_ NSs and the SiO_2_/Si substrate, a layer (marked with “B” in Figure 4a) containing Mo, S and O (37–55 nm) was detected by EDS (Figure S2, Supporting Information File 1). This indicates that partial sulfurization of the initial Mo film (50 nm thick) occurred in the entire volume.

### Field-emission results

**Figure 5:**
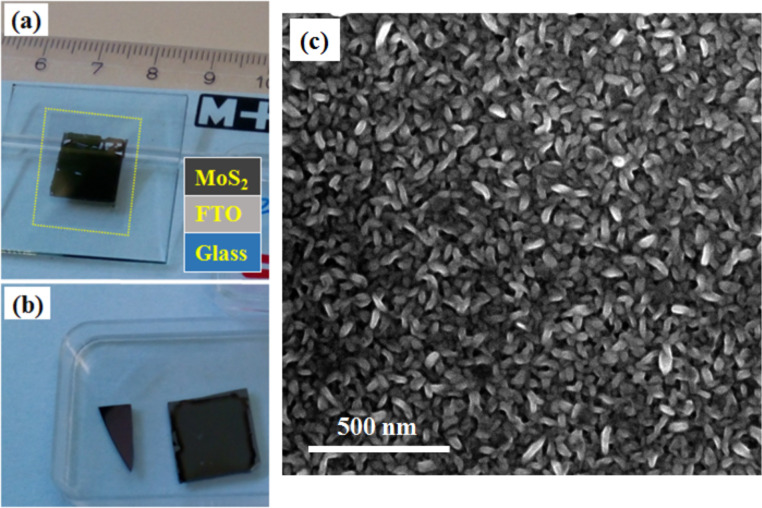
Photographs of (a) wet chemically transferred MoS_2_ sample on the FTO/glass substrate, the FTO film on the glass substrate is marked with a yellow dotted square. (b) The remaining square Si sample after being detached from the MoS_2_ NSs after the buffered oxide etchant. The triangular sample in panel (b) is a piece from the original sample kept for TEM measurements.

FE measurements on the transferred MoS_2_ NSs are given in Figure 5. The transferred NSs were checked using SEM (Figure 5c) and Raman spectroscopy (not shown as it is identical to the as-grown NSs). It was thus confirmed that they are of similar quality to that of as-grown NSs. The Fowler–Nordheim (F–N) equation [28] was modified for a cathode with nanometric field emitters as follows:

[1]
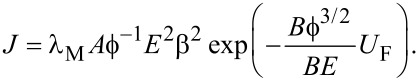


**Figure 6:**
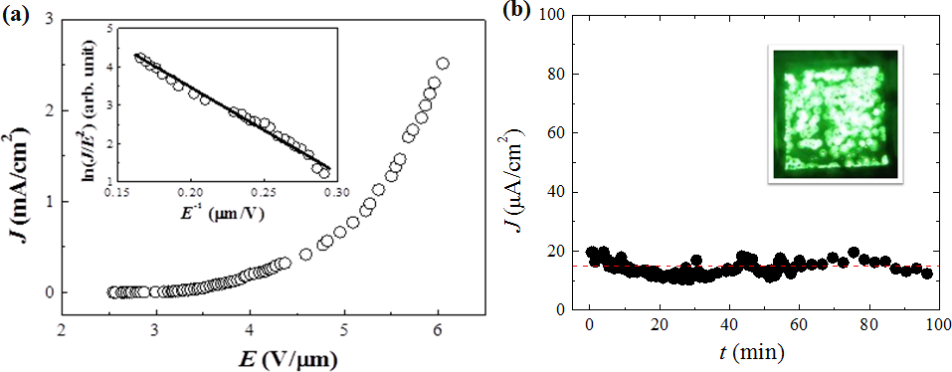
(a) Field-emission current density as a function of the electric field for the transferred MoS_2_ NSs on FTO/glass. The inset shows the corresponding FN plot with a linear fitting used for estimating the field-enhancement factor. (b) The long-term field-emission stability for the same sample at the pressure of ca. 10^−6^ mbar with luminance from the sample.

*E* = *U*/*d*, where *U* is the voltage applied between the flat cathode and the anode screen and *d* is the distance in between (220 µm). *A* and *B* are constants (*A* = 1.54 × 10^−6^ AeV·V^−1^, *B* = 6.83 × 10^6^ eV^−3/2^·Vnm^−1^) that depend on the surface structure. λ_M_ is a macroscopic pre-exponential correction factor. *U*_F_ (the correction factor) is a particular value of the principal Schottky–Nordheim barrier function *U*. β is the local electrical field enhancement factor. 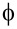 is the work function of the emitter (considered to be 4.04 eV here [29]). In Figure 6a, the current density versus electric field (*J–E*) curve of the transferred MoS_2_ on the FTO sample is displayed. The ln(*J*/*E*^2^) versus 1/*E* graph of the emission data is shown in the inset of Figure 6a. The linear behavior indicates that FE from the NSs is dominated by the tunneling effect. It can be seen that the density of emission current increases rapidly along with the increasing of the applied electric field (Figure 6a). The turn-on field, defined at a current density of 10 µA/cm^2^, and the threshold field at 1 mA/cm^2^, are 3.1 and 5.3 V/µm, respectively. These data are found to be better than the previously reported values for MoS_2_ nanoflowers [13] or multilayered MoS_2_ [8,12], and comparable with the vertically aligned MoS_2_ NSs with ultrathin edges in [9] (Table 1). It has been shown previously that vertically grown 1D nanowires/nanotubes and 2D NSs with atomically thin edges may considerably improve the FE properties, so that vertically standing materials are promising for FE applications [30]. The low turn-on field and threshold field of the MoS_2_ NSs could be due to their vertically aligned extremely thin edges, forming a nano-tip-like structure (Figure S1, Supporting Information File 1).

**Table 1:** Comparison of field-emission properties of MoS_2_ nanosheets (NSs) produced using different methods.

morphology	growth method	turn-on field (V/μm) @ 10 μA/cm^2^	threshold field (V/μm) @ 1 mA/cm^2^	maximum current density *J*_max_ (mA/cm^2^)	enhancement factor β	FE measurement pressure (mbar)	year/reference

nanoflowers composed of NSs	CVD	4.5–5.5	6.2–7.0	50	572–700	10^−7^	2003 [13]
planar (with a few protruding) NSs	hydrothermal	3.5	NA	0.9	1138	10^−8^	2013 [12]
agglomerated NSs	hydrothermal	13.2	NA	0.09	<500	10^−8^	2015 [8]
vertically aligned sparsely distributed NSs	CVD	2.5	NA	0.2	6240	10^−6^	2016 [9]
vertically aligned densely distributed NSs	CVD	3.1	5.3	>2.5	856	10^−6^	2018 [current work]

A considerable enhancement in FE can be achieved by tuning the geometrical morphology of the emitter surface and thus it is important to control the surface morphology for producing better field emitters [9,30–31]. The emitter surface is rough for nanomaterials deposited as a planar cathode and, therefore, for a given emission site the applied electric field varies from the local electric field. The ratio of the actual local electric field to the applied average electric field is known as the field-enhancement factor. In the present case, the field-enhancement factor, commonly used for evaluating FE properties, is calculated from the slope *m* of the F–N plot (a plot of ln(*J*/*E*^2^) versus 1/*E* as given in the inset of Figure 6a, using the following equation:

[2]
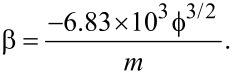


A value of 867 is estimated by linearly fitting the FN plot. This value is indeed higher than that of MoS_2_ nanoflowers [8,13], and in the range of few-layered MoS_2_ NS field emitters (β = 1138 for hydrothermally produced MoS_2_ NSs) [12]. The enhancement factor for our NSs is apparently smaller than the one recently reported on vertically aligned MoS_2_ NSs (β = 6240 for NSs with ultrathin edges) [9]. However, the electric field required to obtain a high current density of 10 µA/cm^2^ is much lower for our as-produced NSs as compared to the above report (Table 1). It can be seen that a high current density ≥2.5 mA/cm^2^ can be achieved (Figure 6a). For the sparsely distributed and ultrathin edge NSs, the current density is only 0.2 mA/cm^2^ [9]. To obtain high current density from the material is very important for practical applications. Thus, as compared with previously published horizontally aligned monolayer or the vertically aligned MoS_2_ NSs or nanoflowers, the as-produced NSs present a clear advantage in terms of low turn-on field and high current density. It has been demonstrated that film morphology of the cathode greatly influences the field-emission performance and the electrons are easier to be extracted from a film with more exposed edges [32]. Hence, we believe that the tip-like geometry (Figure 1d) and exposed edges (Figure 4) of the MoS_2_ NSs are enhancing the tunneling probability for electrons in layered nanomaterials. A similar effect has been observed previously in carbon nanotubes [33].

The emission current density versus time plot is shown in Figure 6b. An almost stable (fluctuations between 10 and 20 µA/cm^2^) emission over a period of 100 min without any measurable degradation can be seen. The densely packed NSs might be playing an important role to achieve high current density and faster heat dissipation, thereby reducing the burning out of active emission sites induced by Joule heating. The as-grown NSs could also possibly be used as heat dissipating nano-channels in FE or electronic devices [34]. Moreover, the excellent luminance uniformity (except over the film area broken during the transfer process, refer to Figure 5a) of the cathode is demonstrated from the inset in Figure 6b. The stability and luminance results indicate that the as-grown material could be a good emitter in vacuum environments relevant to the industry.

## Conclusion

Uniform and continuous MoS_2_ NSs of 5–20 nm thickness were successfully grown by CVD on SiO_2_/Si substrates by a double sulfurization step. Micro-Raman and XPS measurements revealed high-quality growth of the vertically aligned NSs. Cross-section TEM measurements revealed that the NSs of 5–20 nm thickness have a height of a few tens of nanometers. AFM measurements showed that the NSs formed a FE nano-tip-like morphology. We have demonstrated that the as-grown NSs can be transferred onto a desired substrate such as conducting FTO/glass employing a wet-chemical transfer process. These NSs show very interesting FE properties at room temperature and in high vacuum (10^−6^ mbar) as proven by the low turn-on field of 3.1 V/μm and low threshold field of 5.3 V/µm. In future, the as-grown NSs could be potentially used for FE and display device applications.

## Supporting Information

File 1 Additional experimental data.
