# Supplementary material for: Toward the use of CVD-grown MoS2 nanosheets as field-emission source
Source: Beilstein J Nanotechnol. 2018 Jun 7;9:1686–94. doi: 10.3762/bjnano.9.160 (PMC6009318; doi:10.3762/bjnano.9.160)
Supplement: File 1 — Additional experimental data. [file Beilstein_J_Nanotechnol-09-1686-s001.pdf]

Supporting Information

for

Toward the use of CVD-grown MoS_2 nanosheets as field-emission source

Geetanjali Deokar*[1,4], Nitul S. Rajput[1], Junjie Li[2], Francis Leonard Deepak[2], Wei Ou-Yang*[3], Nicolas Reckinger[4], Carla Bittencourt[5], Jean-Francois Colomer[4] and Mustapha Jouiad*[1]

Address: [1]Department of Mechanical and Materials Engineering, Masdar Institute of Science and Technology, A part of Khalifa University of Science and Technology, 54224, Abu Dhabi, United Arab Emirates; [2]Department of Advanced Electron Microscopy, Imaging and Spectroscopy, International Iberian Nanotechnology Laboratory (INL), Avenida Mestre Jose Veiga, Braga 4715-330, Portugal; [3]Engineering Research Center for Nanophotonics & Advanced Instrument, Ministry of Education, School of Physics and Materials Science, East China Normal University, 3663 North Zhongshan Road, Shanghai 200062, China; [4]Research Group on Carbon Nanostructures (CARBONNAGe), University of Namur, 61 Rue de Bruxelles, 5000 Namur, Belgium and [5]Chimie des Interactions Plasma-Surface (ChIPS), CIRMAP, Research Institute for Materials Science and Engineering, University of Mons, Mons, Belgium

Email: Geetanjali Deokar* - gdeokar@masdar.ac.ae;

Wei Ou-Yang* - ouyangwei@phy.ecnu.edu.cn;

Mustapha Jouiad* - mjouiad@masdar.ac.ae

*Corresponding author

Additional experimental data

Table S1: Peak fitting analysis results of the Mo 3d, S 3p, and O 1s core-level high-resolution XPS spectra from Figure 2b,c (BE = binding energy and FWHM = full width at half maximum).

Phase	Core level Mo3d / S1s	Peak BE	FWHM	Core level S2p/O1s	Peak BE	FWHM
MoS_2	$Mo3d_{5/2}$	228.9	0.81	$S2p_{3/2}$	161.8	0.73
	$Mo3d_{3/2}$	232	0.91	$S2p_{1/2}$	163	0.73
MoS_2	S2s	226.2	2.02			
MoO_2	$Mo3d_{5/2}$	232.7	0.68	O1s	530.3	1.36
	$Mo3d_{3/2}$	235.6	1.67			
-C-O				O1s	532.0	2.54

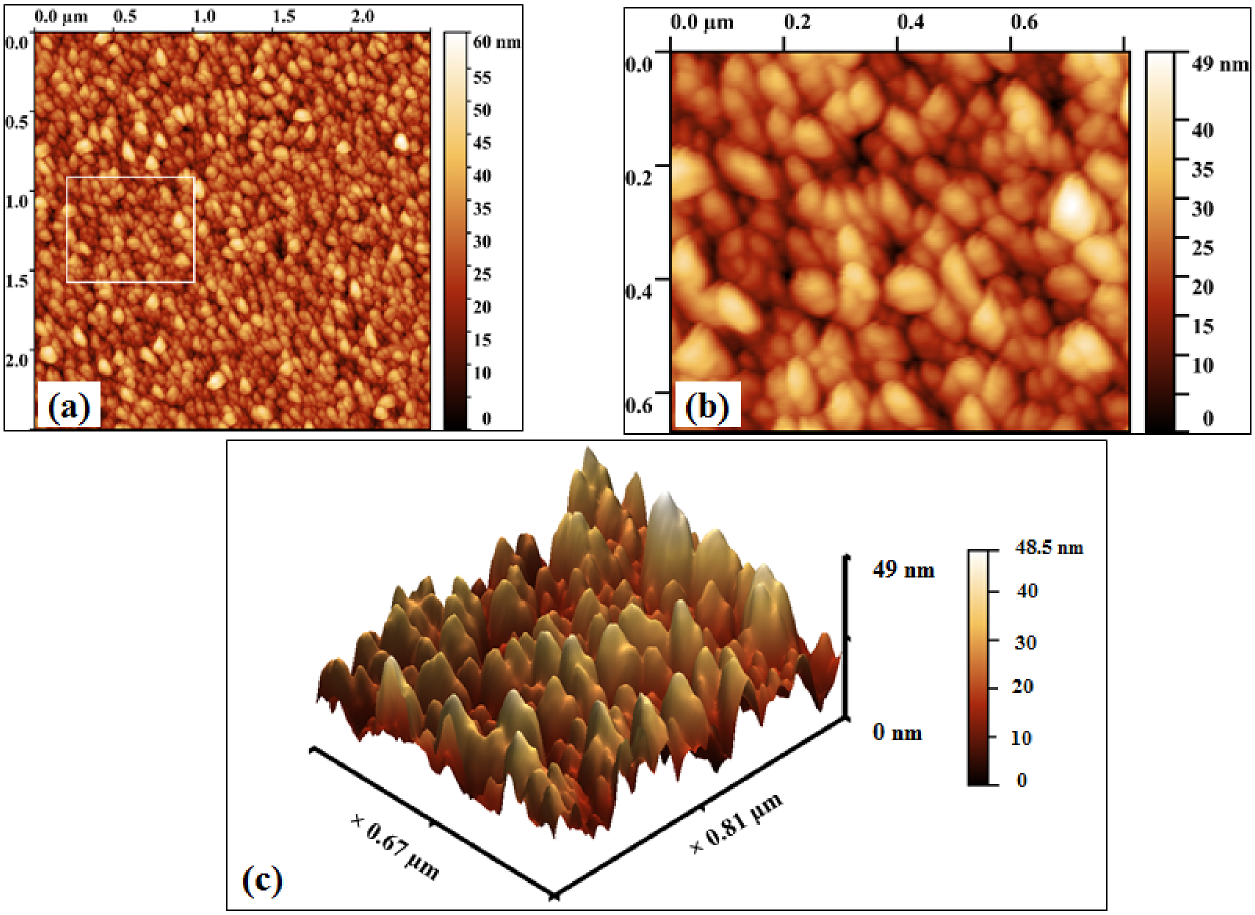

Figure S1: AFM resonant-mode mapping for MoS_2 sample grown by double sulfurization of a 50 nm Mo film at 850 °C on SiO_2/Si substrates: (a) Topographic view of a 2.5 × 2.5 µm^2 area and (b) zoom-in over the white square in panel (a); (c) 3D image of panel (b).

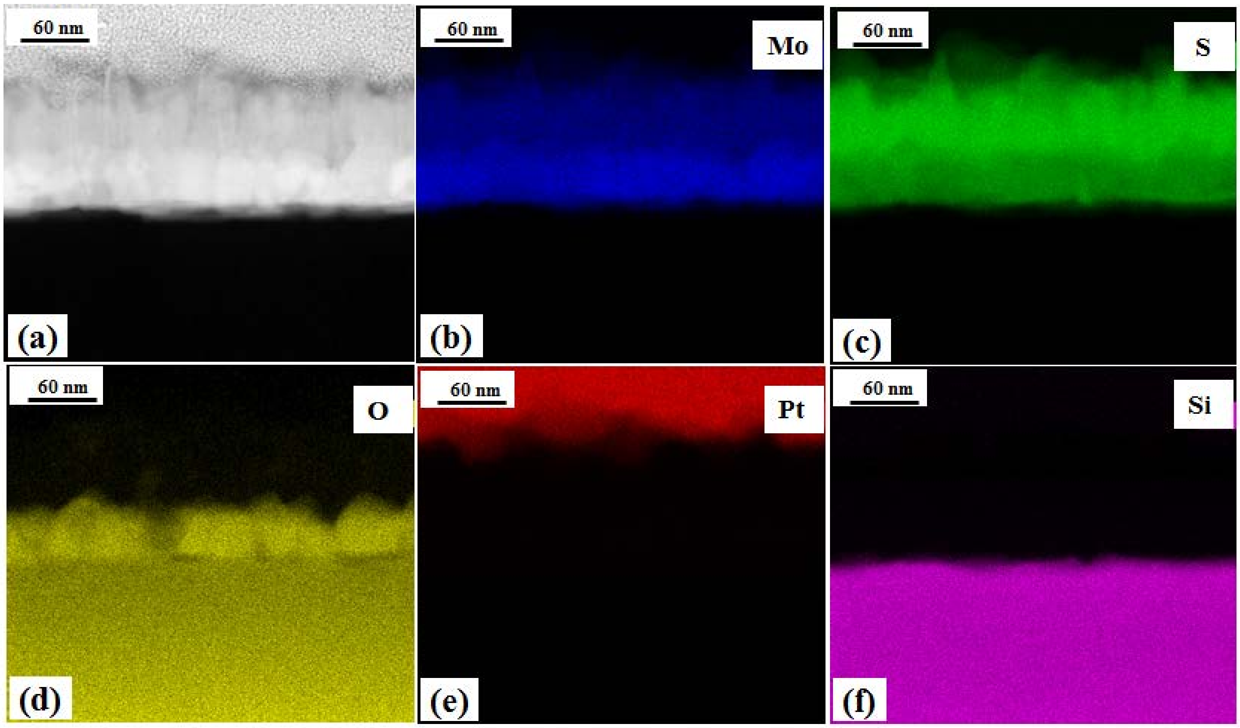

Figure S2: MoS_2 sample grown by double sulfurization of a 50 nm Mo film at 850 °C on SiO_2/Si substrates: (a) HAADF-STEM image, (b–f) EDS mapping results for various elements, over the area in panel (a).
